# Nocardia in an Immunocompetent Patient Simulating Pulmonary Carcinoma: A Case Report and Literature Review

**DOI:** 10.7759/cureus.64491

**Published:** 2024-07-13

**Authors:** Jhon Edwar Garcia Rueda, Karen Yohana García Rueda, Angélica María Bermúdez Flórez, Laura Andrea Peña Mejía, Alejandro Cardona Palacio, Wilfredy Castaño Ruiz

**Affiliations:** 1 Internal Medicine, Universidad Pontificia Bolivariana, Medellin, COL; 2 College of Medicine, Corporacion Universitaria Remington, Medellin, COL; 3 Medical Physics, Universidad de Antioquia, Medellin, COL; 4 Pathology, Universidad de Antioquia, Medellin, COL; 5 Pathology, Universidad Escuela de Ingeniería de Antioquia, Medellín, COL; 6 Thoracic Surgery, Hospital Pablo Tobón Uribe, Medellin, COL

**Keywords:** atypical infection, nocardia spp, nocardia as lung cancer, infection mimicking malignancy, carcinoma lung

## Abstract

Nocardiosis is an opportunistic infectious pathology of low incidence that usually affects the lungs, skin, and brain. It has been implicated in causing serious and potentially fatal infections without treatment. It affects immunocompetent and immunocompromised patients. In immunocompetent patients, it is presented with local conditions, and in immunocompromised patients, it is seen in disseminated forms. We present the case of a 61-year-old male immunocompetent patient with a high suspicion of pulmonary carcinoma, in whom pathology showed infection by *Nocardia spp.*

## Introduction

*Nocardia* is a gram-positive, aerobic, weakly acid-fast, intracellular bacterium of low virulence whose usual environment is soil and water [1]. The order is *Actinomycetales*, and the family is *Nocardiaceae*. The most common species are *Nocardia asteroides*, *Nocardia farcinica*, and *Nocardia brasiliensis*. It has been reported on all continents, all ages, and ethnic groups [2,3].

Nocardiosis is a rare bacterial infection, with a reported incidence of 0.45 per 100,000 inhabitants; up to 60% of cases are associated with immunosuppression [2]. It occurs mainly in patients with cellular immunity deficits, such as people with solid organ transplantation, malignancy, diabetes mellitus, HIV infection, autoimmune diseases, and prolonged therapy with corticosteroids [4,5].

Infections in immunocompetent patients are rare and represent a diagnostic challenge given the clinical and radiological similarity with infectious diseases such as tuberculosis, fungal infections, and neoplasms. We present the case of a male patient with no history of immunocompromised disease with a pulmonary condition simulating primary lung carcinoma.

## Case presentation

A male patient, 61 years old, with no pathological history of importance, an engineer in an asphalt and stone crushing plant, and a recent trip to an amethyst mine in Brazil, presented a clinical picture of three months of evolution that began with subjective fever, chills, nocturnal diaphoresis, and an objective weight loss of 3 kg. Subsequently, he started with dyspnea, hemoptysis, and an objective fever, for which he consulted a local hospital. Initial studies were performed, including a normal chest CT scan with a report of left basal pneumonia, three *bacilloscopies*, and PCR for *Mycobacterium tuberculosis*, which were negative, negative COVID-19 PCR, and negative blood cultures. Treatment was started with 3 g of ampicillin/sulbactam every 6 hours intravenous (IV) and 500 mg of clarithromycin every 12 hours IV for seven days. At the end of treatment, the patient achieved partial improvement in his symptoms.

Two days later, he presented with symptomatic relapse, so he decided to consult another institution, where he was prescribed 500 mg/125 mg amoxicillin/clavulanic acid every 12 hours orally for five days and a tomographic control that reported a pulmonary mass of soft tissue density of 53 x 38 mm in the axial plane, at the apical level of the left upper lobe, contacting the homolateral pleura associated with retraction of the adjacent parenchyma and surrounding areas of ground glass. Due to worsening hemoptysis, he decided to consult a hospital of a higher level of complexity.

**Figure 1:**
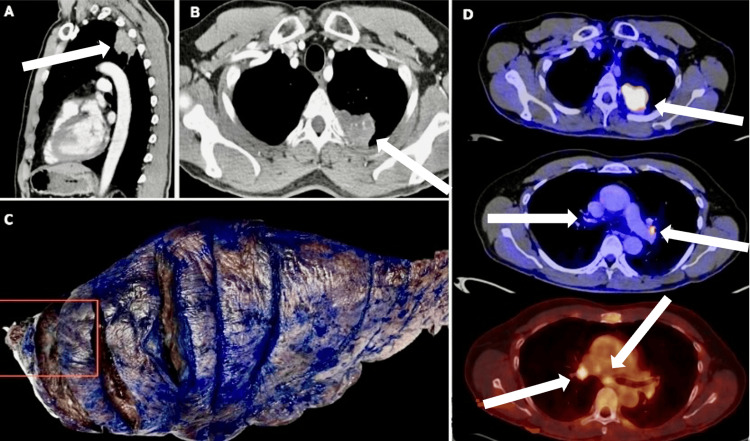
A, B: Contrast tomography of the thorax Mass with spiculated margins in the left upper lobe apicoposterior segment measuring approximately 42 x 32 x 45 millimeters with infiltration to the third rib. C: Lobectomy with costal bone condition. Rectangles indicate the thoracic wall. D: PET-CT: Pulmonary mass with SUVmax of 35.91 in contact with the major fissure and with the parietal pleura, with obliteration of the extrapleural fat, up to where the metabolic activity extends. Mediastinal adenopathies with an SUVmax of 7.45 are located in both the pulmonary hilar, paraaortic, and subcarinal regions.

On admission, HIV, hepatitis B, and C infections were ruled out under clinical suspicion of an infectious or oncologic condition. A contrasted chest CT scan was performed, which reported mediastinal adenopathies and a mass in the left upper lobe apicoposterior segment with spiculated margins measuring 42 x 32 x 45 mm with infiltration to the third rib (Figures 1A, 1B). A contrasted brain MRI was performed, which was negative for metastasis, and PET-CT, which reports a solid pulmonary mass with an SUVmax of 35.91 in contact with the major fissure and the parietal pleura, with obliteration of the extrapleural fat, to where the abnormal metabolic activity extends, as an early sign of invasion of the thoracic wall. Mediastinal adenopathies with an SUVmax of 7.45 are located in both the pulmonary hilar, paraaortic, and subcarinal regions. The above suggests a T3 classification (invasion of the lesion to the thoracic wall, third rib), N3 (station 4R and positive), and M0, obtaining a stage IIIC (Figure 1D). ﻿

A CT-guided biopsy was performed and was negative for malignancy. Consequently, invasive staging was ordered by video-assisted mediastinoscopic lymphadenectomy (VAMLA) with evidence of large posterior mass invading ribs, hilar lymphadenopathy, great adherence to tissue in the aortopulmonary window, and invasive station 5 lymphadenopathy involving the left recurrent laryngeal nerve. Lymph nodes were obtained (stations 2L, 2R, 4L, 4R, 7, 10L, 10R), all with negative histology for malignancy, so it was reclassified as T3N0M0-IIb.

It was decided to perform video-assisted thoracoscopy plus frozen section biopsy where glands with reactive changes secondary to extensive necrosis favoring adenocarcinoma were observed, so a left upper pulmonary lobectomy was performed, with involved costal resection and mediastinal lymphadenectomy stations 4L, 5, 6, 8, 9.

**Figure 2:**
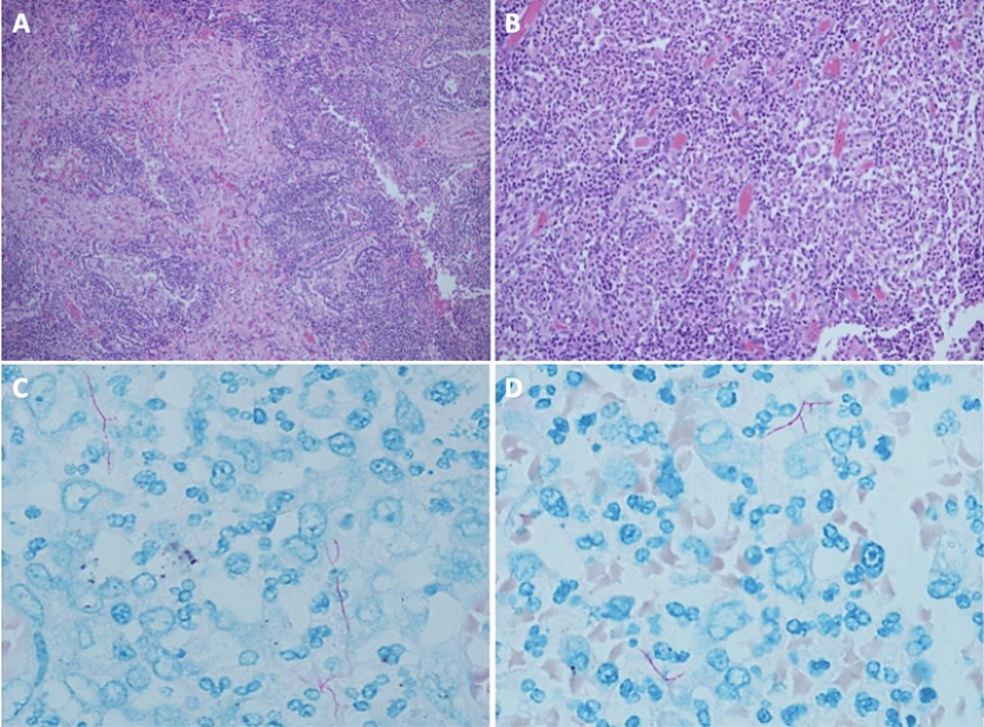
Microscopic findings A, B: Abscessed liquefactive necrosis. Hematoxylin-eosin staining is 40x. C, D: ZN modified for nocardia. Positive for filamentous structures. 40x.

A surgical sample from a pulmonary lobectomy was sent to the pathology department, weighing 278 grams and measuring 26 x 16 x 4 cm. The cut identified a necrotic lesion of 4 x 4 x 4 x 4 cm in contact with the costal surface (Figure 1C). The entire sample was processed, and the microscopic findings showed an altered pulmonary architecture with extensive areas of liquefactive necrosis composed of abundant neutrophilic polymorphonuclear neutrophils forming microabscesses (Figures 2A, 2B).﻿

Special Ziehl-Neelsen (ZN), modified ZN, methenamine silver, and periodic acid-Schiff (PAS) stains were requested, negative for acid-fast *bacilli* and fungal structures; additionally, a ZN for *Nocardia* was requested, which was positive for multiple filamentous structures morphologically compatible with *Nocardia spp.* (Figures 2C, 2D).

The patient was diagnosed with pulmonary nocardiosis, and treatment was started for eight weeks with 500 mg Imipenem every 6 hours IV and 320/1600 mg Trimethoprim/Sulfamethoxazole (TMP/SMX) orally every 8 hours, with an adequate evolution and response. After two months of hospitalization, he was discharged with 320/1600 mg of TMP/SMX orally every 12 hours for six months.

In the outpatient follow-up by infectology, he successfully completed the treatment, with a remission of symptoms. In addition, he reported that his mother required enucleation eight years ago for ocular and pulmonary nocardiosis.

## Discussion

*Nocardia* is an intracellular bacterium characterized by a filamentous growth pattern, despite being a weakly alcohol-resistant acid-fast bacillus; it is positive for ZN and modified ZN histochemical staining. Predisposing risk factors are cancer, HIV infection, prolonged steroid treatment, chronic obstructive disease, diabetes mellitus, and alcoholism [4,6-8].

Its pathogenesis is not yet fully understood and continues to be the subject of study; however, it is believed to be related to the ability to prevent phagocytosis by inhibiting phagosome and lysosome fusion, resisting the microbicidal actions of phagocytes by blocking phagosome acidification, and resistance to respiratory burst by the release of superoxide dismutase [9-13].

The most common condition caused by *Nocardia* is pulmonary and occurs in 50-70% of cases, originating from the inhalation of bacteria. However, it can spread to any organ, with conditions described in the eyes, kidneys, bones, and skin. The latter occurs after inoculation by trauma [7]. *Nocardia* has a tropism for the central nervous system, where it produces multifocal abscesses and signs of increased intracranial pressure, so it has come to be confused with brain metastases of a supposed pulmonary adenocarcinoma, where the etiology found is disseminated nocardiosis. Table 1 shows the case reports of *Nocardia* simulating pulmonary carcinoma found in the literature [8-12, 14-18].

**Table 1:** Reports of Nocardia simulating lung carcinoma G: Gender, F: Female, M: Male, A: Age, TMP/SMX: Trimethoprim/sulfamethoxazole, DM: Diabetes mellitus, HBP: High blood pressure, AF: Atrial fibrillation, COPD: Chronic obstructive pulmonary disease, CVA: Cerebrovascular accident, IV: Intravenous, O: Orally, LUL: Left upper lobe, RLL: Right lower lobe, RML: Right middle lobe, N: Nocardia.

DOI	Tomographic findings in the thorax	G	A	Treatment	Initial symptoms	Species	History
[8]	Multiple bilateral subpleural soft tissue densities in lower lobes, calcification, and cavitation in some lesions.	F	55	TMP/SMX	Fever, cough with sputum, and hemoptysis for 2 weeks.	N. otitidiscaviarum	Treated pulmonary tuberculosis.
[9]	Mass of 45 mm in LSI, upper segment (S1+2), and mediastinal lymph nodes. The mass showed contrast enhancement and necrosis was suspected.	M	76	Meropenem followed by TMP/SMX and levofloxacin.	Persistent cough, sputum production and chest discomfort.	N. exalbida	Overactive bladder, hemorrhoids and smoking (60 packs/year).
[10]	Lung mass in the right middle lobe, multiple bilateral pulmonary nodules, as well as pneumonia in the RLL.	M	55	TMP/SMX and meropenem IV.	Productive cough, headache, hyporexia and emesis. Weight loss (15 kg) during the last year.	N. cyriacigeorgica	HBP, hyperlipidemia, migraine, COPD and smoking (30 packs/year).
[11]	Centrally well-defined hypodense lesion, 6 cm, in RML, S5 segment in contact with the parietal pleura, the right dome of the diaphragm, and pericardium, without adenopathy.	M	70	Sulfamethoxazole and ceftriaxone.	Persistent fever and cough.	N. cyriacigeorgica	No history.
[12]	Irregular lung mass in the right lower lobe, measuring 6.04 x 5.21 x 3.82 cm, heterogeneous, with spiculated edges and small cavitations inside, mediastinal adenopathies.	M	73	TMP/SMX: 15 mg/kg per day IV for 4 weeks. End with TMP/SMX O for 6 months.	Persistent cough and hemoptotic sputum of 6 months of evolution.	Nocardia spp.	Ex-smoker (45 packs/year), hypertension, stroke and anxiety.
[15]	Lung nodules.	F	72	TMP/SMX and moxifloxacin for 6 months.	Aphasia and weakness of the right hemibody.	N. brasiliensis	DM, HBP, sinus node dysfunction, pacemaker, stroke, and AF.
[16]	Mass in the apical segment of the upper lobe of the right lung and multiple parenchymal lesions.	M	39	Ampicillin sulbactam and clarithromycin for 14 days.	Recurrent fever and cough with purulent sputum for two months.	No report	Neuro-Behcet ¸ 2 months ago on immunosuppressive treatment.
[17]	Right lung mass in lower lobe, consolidation and pleural effusion.	F	40	TMP/SMX for 2 months.	Cough and hemoptysis of 3 months of evolution.	No report	Renal failure.
[18]	Infiltrating mass in the left lobe and nodules in the left lobe.	F	74	TMP/SMX for 6 months.	Fatigue, fever, chills, productive cough, weight loss.	No report	Smoker, COPD, requiring oxygen

Pulmonary disease can be subacute or indolent. Clinically, pulmonary nocardiosis is indistinguishable from pneumonia caused by other bacterial agents, which makes it difficult to have suspicion in the immunocompetent population. It may present with pictures of pneumonia with symptoms of cough with or without purulent sputum, fever, and weight loss. The pathologic findings in our case are consistent with those found in the literature, where the pathologic features reported are necrotizing abscesses and the formation of granulomas, usually containing polymorphonuclear macrophages and lymphocytes [13,14].

The imaging findings of pulmonary nocardiosis are not well described in the literature; its radiological appearance is variable, which can generate confusion at the time of diagnosis with other entities [12]. In relation to chest tomography, the series of Tsujimoto et al. describes the presence of nodules at 32.5%, cavitation at 30%, consolidation at 22.5%, and masses at 12.5%, with no report of adenopathy, while the series of Liu et al. reported consolidation at 88.9%, masses or nodules at 66.7%, and only two patients with adenopathy [9].

Other case reports have evidenced the presence of pulmonary lesions with amorphous calcifications, cavitations, and adenopathies that increase the suspicion of neoplasia [9]. Our patient presented infrequent radiological findings, which, together with mediastinal adenopathies in CT and PET-CT, made it necessary to make the differential diagnosis of lung cancer. As a nexus, we found exposure to volatile hydrocarbon elements, crushed stones, and a trip to an amethyst mine in another country. In addition, at the time of diagnosis, the patient reported that his mother was treated for pulmonary and ocular nocardiosis with enucleation eight years ago when he lived with her.

Nocardiosis, due to its systemic symptoms added to pathology and imaging findings, could simulate a malignant and even metastatic disease. Due to the great clinical and radiological similarity with tuberculosis, non-tuberculous mycobacteria, fungal infections, and even neoplasms, this diagnosis is a great challenge. 

## Conclusions

Nocardiosis is a rare opportunistic infection, usually affecting immunocompromised patients. Symptoms and radiological studies are not specific, which makes its diagnosis difficult. It should be considered in patients with suspected neoplasia when the clinical presentation is unusual and pathological studies are inconclusive. Clinical evaluation, laboratory and histological examinations, and adequate correlation with imaging findings are the appropriate approaches to diagnosis. Prompt antibiotic treatment is crucial for treatment and to avoid dissemination.
